# Anxiety, Depression and Post Traumatic Stress Disorder after critical illness: a UK-wide prospective cohort study

**DOI:** 10.1186/s13054-018-2223-6

**Published:** 2018-11-23

**Authors:** Robert Hatch, Duncan Young, Vicki Barber, John Griffiths, David A. Harrison, Peter Watkinson

**Affiliations:** 1 NIHR Academic Clinical Fellow in Intensive Care Medicine, Oxford Deanery, Oxford, UK; 2 Professor of Intensive Care Medicine, University of Oxford, Oxford, UK; 3 OCTRU Hub Manager, University of Oxford, Oxford, UK; 4 Consultant in Intensive Care Medicine, Oxford University Hospitals NHS Foundation Trust, Oxford, UK; 5 Head Statistician, Intensive Care National Audit & Research Centre (ICNARC), London, UK; 6 Associate Professor of Intensive Care Medicine, Oxford University Hospitals NHS Trust, Oxford, UK; 7 Kadoorie Centre for Critical Care Research and Education, University of Oxford, Level 3, John Radcliffe Hospital, Headley Way, Oxford, OX3 9DU UK

**Keywords:** Anxiety, Depression, Post-traumatic stress disorder, PTSD, Post-traumatic stress disorder civilian checklist, PCL-C, Post-Intensive Care Syndrome, PICS, Questionnaire, Postal, Intensive care, Critical care, Survivors, Multicentre study, Critical illness, Outcome assessment

## Abstract

**Background:**

Survivors of intensive care are known to be at increased risk of developing longer-term psychopathology issues. We present a large UK multicentre study assessing the anxiety, depression and post-traumatic stress disorder (PTSD) caseness in the first year following discharge from an intensive care unit (ICU).

**Methods:**

Design: prospective multicentre follow-up study of survivors of ICU in the UK.

Setting: patients from 26 ICUs in the UK.

Inclusion criteria: patients who had received at least 24 h of level 3 ICU care and were 16 years of age or older.

Interventions: postal follow up: Hospital Anxiety and Depression Score (HADS) and the Post-Traumatic Stress Disorder (PTSD) Check List-Civilian (PCL-C) at 3 and 12 months following discharge from ICU.

Main outcome measure: caseness of anxiety, depression and PTSD, 2-year survival.

**Results:**

In total, 21,633 patients admitted to ICU were included in the study. Postal questionnaires were sent to 13,155 survivors; of these 38% (4943/13155) responded and 55% (2731/4943) of respondents passed thresholds for one or more condition at 3 or 12 months following discharge. Caseness prevalence was 46%, 40% and 22% for anxiety, depression and PTSD respectively; 18% (870/4943 patients) met the caseness threshold for all three psychological conditions. Patients with symptoms of depression were 47% more likely to die during the first 2 years after discharge from ICU than those without (HR 1.47, CI 1.19–1.80).

**Conclusions:**

Over half of those who respond to postal questionnaire following treatment on ICU in the UK reported significant symptoms of anxiety, depression or PTSD. When symptoms of one psychological disorder are present, there is a 65% chance they will co-occur with symptoms of one of the other two disorders. Depression following critical illness is associated with an increased mortality risk in the first 2 years following discharge from ICU.

**Trial registration:**

ISRCTN Registry, ISRCTN69112866. Registered on 2 May 2006.

**Electronic supplementary material:**

The online version of this article (10.1186/s13054-018-2223-6) contains supplementary material, which is available to authorized users.

## Background

Survivors of critical illness are at risk of experiencing significant physical, cognitive and psychological issues after discharge [1]. New or worsening impairment in physical, cognitive or mental health status following treatment on an intensive care unit (ICU) is known as post-intensive care syndrome (PICS) [2]. The psychopathological components of PICS are estimated to occur in up to a third of survivors of ICU treatment [1]. The major psychological conditions described are anxiety, depression and post-traumatic stress disorder (PTSD).

Symptoms of anxiety following critical illness occur in 25–46% of patients in the 3–14 months following discharge from an ICU [3]. Post ICU anxiety is associated with psychiatric symptoms, memories and delusions [4–6]. Unlike the general population, where anxiety is more common in women aged 30–44 years [7], no association with age, sex, disease severity or length of stay has been found in patients treated on an ICU. This difference may reflect a causal association or the underpowered nature of the individual studies (where the largest previous study included 255 patients [8]).

Depressive symptoms occur in around 29% of survivors at 3, 6 and 12 months post ICU discharge [3]. Depressive illness following intensive care is associated with symptoms of psychological distress (anxiety, stress and anger). The Hospital Anxiety and Depression Scale (HADS) is the most commonly used (and validated) tool; however, the most recent meta-analysis identified only 387 unique patients with HADS data at 3 and 6 months post discharge and a further 412 at 6 and 12 months [9]. Like anxiety, there was no association with age or sex, in contrast to findings in general populations [9]. In addition, there was no correlation with ICU length of stay or illness severity.

A recent meta-analysis (2015) estimated PTSD symptom prevalence at 17–34% at 12 months post-ICU discharge [3]. Risk factors for the development of post-ICU PTSD include the presence of pre-ICU anxiety and depression.

The risk of mortality in survivors of critical illness is 3.4 times greater than that of the general population over the 5 years following discharge [10]. Mortality remains greater than that of the general population for up to 4 years following ICU admission. There is a known association between depression and an increased risk of death, both in the general population [11, 12] and specific subgroups of patients with illness-comorbid depression. Conversely, anxiety and/or PTSD have been associated with a decreased mortality hazard [11]. Whether the presence of psychopathology affects mortality following treatment on an ICU overall is unknown.

Previous studies of psychopathology after critical illness are limited by their size. In many cases they are performed in specific sub-populations, conditions or as part of a randomised controlled trial (RCT) [3, 9, 13]. Survivors of critical illness are heterogeneous in terms of illness severity, treatment and long-term prognosis. Few studies have concurrently assessed symptoms of anxiety, depression and PTSD in a general adult ICU population. ICU-specific conditions such as acute respiratory depression syndrome (ARDS) are associated with poorer longer-term outcome and a high incidence of psychopathology [13]. However, these diagnoses represent the minority of overall ICU admissions.

### Objective

We postulated that illness-comorbid depression is independently associated with survival, when other known associations such as age and illness severity are considered. We undertook a large multicentre postal survey of all patients admitted to participating UK general adult ICUs. Our objective was to describe the pattern of psychopathology occurring in survivors at 3 and 12 months following discharge from ICU and to assess any association with mortality.

## Methods

This study is reported following the Strengthening of Reporting in Observational studies in Epidemiology (STROBE) guidance [14].

### Study design

The Intensive Care Outcomes Network study (ICON) was a UK multicentre prospective cohort study assessing heath related quality of life (HRQoL) and caseness of anxiety, depression and PTSD, by postal questionnaire, following at least 24 h treatment on an ICU. Caseness is the degree to which the accepted standardised diagnostic criteria for a given condition are applicable to a given patient. A questionnaire study assesses the self-reported symptom burden consistent with a specific disorder but cannot be considered diagnostic. The trial was registered ISRCTN69112866 (assigned 2 May 2006) and the study protocol has been published [15]. This study was conducted in three consecutive phases (summarised in Additional file 1). Ethical approval for phases 1 and 2 was granted by Oxfordshire Research Ethics Committee B (REC 06/Q1605/17). Ethical approval for phase 3 was granted by National Research Ethics Service – South Central Berkshire (REC 11/SC/0172). The ICON study had Section 60 of the Health and Social Care Act 2001 (subsequently Section 251 of the National Health Service (NHS) Act 2006) approval (PIAG 2–05(e)/2006). This granted permission to record details of all admissions meeting the inclusion criteria at participating ICUs.

### Setting

Phases 1 and 2 took place in the same 26 UK ICUs (1 university hospital, 6 university-affiliated hospitals and 19 district general hospitals) and postal questionnaires were sent at 3, 12 and 24 months following discharge form ICU. Phase 1 recruited from November 2006 to May 2008 and recruited 9582 patients. Phase 2 recruited from May 2008 to October 2010 and recruited 18,490 patients as part of an RCT to study the effect of different questionnaire burden on response rate, the results of which have been published [16].

Phase 3 took place between May 2012 and May 2013 in 31 UK ICUs (10 university hospital, 3 university-affiliated hospitals and 18 district general hospitals), and of these 18 recruiting centres are common with phases 1 and 2. Phase 3 recruited 2876 patients, with postal questionnaires administered at 3 and 12 months post ICU discharge. Questionnaire burden was identical to phase 1. Where possible patients were approached by a research nurse prior to their discharge from hospital. Patients were followed up by telephone if they later failed to respond to a postal questionnaire.

### Participants

We extracted data from all three phases of the ICON study database, with the exception of group A from the second phase, as this group did not receive psychological instruments by design [15, 16]. Each phase had identical inclusion and exclusion criteria.

Eligible patients received level 3 care on an ICU (as defined by the Intensive Care Society, London [17]), for at least 24 h and were 16 years or older at ICU admission. We excluded patients not registered with a general practitioner or of no fixed abode (factors anticipated to prevent follow up in the study). We also excluded patients taking part in another questionnaire follow-up study run by the same research office and in phase 3, those patients who withdrew consent prior to discharge from hospital (as we could not legitimately track their mortality even to hospital discharge). In addition, we excluded those patients who could not be matched to the Intensive Care National Audit & Research Centre (ICNARC) Case Mix Programme database and patients who were not captured by the ICON study during their incident admission to ICU during their hospital stay (as we were timing questionnaire response from their first exposure to ICU). Patients could withdraw their consent at any point during any phase of the study (by contacting the study office or by returning the survey blank). This resulted in their personal identifying data being purged from the study database, anonymising their record at that point. We did not contact patients following a specific request by their GP.

### Variables

Patients received a letter introducing the study at ICU discharge. The letter explained that they might receive mail from the study team and provided contact details for the study office. Eligible patients received postal questionnaires at 3 and 12 months following discharge from ICU. Each mailing included the HADS (14 questions, 7 depression and 7 anxiety, each scored ordinally 0–3), the Post Traumatic Stress Disorder Check List – Civilian version (PCL-C – 17 questions score ordinally 1–5) and other health related quality of life instruments (see Additional file 1). When there was no response to the first mailing, this was followed by a second postal copy 14 days later. In phases 1 and 2 no response after the second mailing was considered a loss to follow up. In phase 3, an additional attempt was made to call the patient.

A cut-off score ≥ 8 for either HADS anxiety or depression scales defined caseness of the respective condition [18]. We applied HADS boundaries for mild, moderate and severe symptoms to those exhibiting caseness [19]. A PCL-C score ≥ 45 defined PTSD caseness [20].

### Data sources and measurement

We linked participant records with the ICNARC Case Mix Programme [21] to obtain admitting diagnoses, severity of illness scores and to capture previous admissions to ICU during their hospital stay. We checked survival and current registered address with the patient’s registered general practitioner (GP) and the National Health Service Summary Care Record [22] before posting each questionnaire pack.

With the exception of those who withdrew consent, we linked participants to the Medical Research Information Service (MRIS) run by NHS Digital. This provided linked mortality data and event notification from the Office of National Statistics (ONS).

### Bias

In phase 1 and 2, clinical staff gave participants a letter explaining that they would receive ICON documentation in the post at the point of discharge from the ICU. In phase 3 a study nurse aimed to visit the patients between ICU and hospital discharge to introduce the ICON study, where a study nurse was available. Further information was provided and a second visit could be arranged to provide written consent if so wished. Patients could remove their consent at any time.

### Study size

Study size was based on the total number of patients meeting inclusion criteria and admitted to the participating units inside the recruitment period.

### Quantitative variables

For this analysis the primary outcome was the proportion of patients meeting predefined thresholds for caseness of anxiety and depression (using the HADS scale) and for PTSD (using PCL-C) at 3 and 12 months following discharge from ICU. Secondary outcome measures were survival at 3, 12 and 24 months following discharge from ICU, the proportion of individual patients transitioning these thresholds between time points and the correlation between PTSD, anxiety and depression.

### Statistical methods

Statistical analysis was undertaken using R Core v3.4.1 [23]. We did not correct for multiple testing. Response was defined as return of a questionnaire with valid written consent. Each instrument was scored in accordance with the author’s instructions. Individual responses not meeting these instructions were considered invalid and excluded from further analysis. The proportions of patients meeting the criteria for caseness were calculated for each instrument at each time point. Those meeting the HADS thresholds were further subdivided by symptom severity (mild, moderate, severe).

Population demographics, responses to the individual psychological instruments and change analysis were presented in keeping with the pre-specified data collection plan for the ICON Study. Survival analysis was first performed using the Kaplan-Meier (KM) method. Patients were right censored when we could no longer track their mortality (lost to follow up). This occurred when patients explicitly withdrew their consent (excluding them from being enrolled with MRIS/ONS). Study phases and participants with and without caseness were compared using the log-rank test with *p* < 0.01.

We performed ad-hoc Cox proportional hazards modelling to study the effects of depression, anxiety and PTSD caseness on survival, adjusting for known confounders. We verified the assumption of proportional hazards prior to conducting the analysis. The study design meant that censoring of individual subjects did not occur as a result of the disease process. We identified age (as a continuous variable), sex (as a binary variable) and severity of illness Acute Physiology and Chronic Health Evaluation (APACHE) II score as a continuous variable [24]) as potential confounders from the literature [3, 13, 25]. The interpretation of a continuous variable is that for each additional unit increase in the continuous variable the hazard ratio increases by the value reported. The interpretation of a binary variable is the hazard ratio associated with male versus female (sex) or caseness versus no caseness for the other variables.

The effect of these variables on survival was confirmed using univariable Cox proportional hazard analysis. Four multivariable models were then constructed. All included the co-founders identified in the literature. Models 1–3 included each psychological disorder in isolation and model 4 included all three.

## Results

### Participants

We screened 21,633 patients for eligibility across the three phases of the study in November 2006 to May 2013: 19,822 patients satisfied the eligibility requirements. Of these, 3289 (17%) died during their admission to ICU. Following discharge alive from ICU, 2710 (2710/19,822 (14%)) of those eligible died in the subsequent 75 days. There were 429 patients (429/13,823 (3%)) excluded from follow up by request of their general practitioner (GP) (see the patient flow diagram in Additional file 2).

Of those who were alive at the point of being sent a questionnaire at 3 or 12 months, 4943 patients (4943/13,155 (38%)) completed at least one survey. Of those mailed at 3 months 4809 (4809/12,777 (38%)) completed a survey. Of those mailed at 12 months, 3569 (3569/4936 (72%)) completed a survey (non-respondents and those who actively declined to consent at 3 months were not mailed). In total 2943 patients (22,943/13,155 (22%)) actively declined to consent (by returning a blank form or contacting the research office at 3 or 12 months).

### Descriptive data

Table 1 shows the demographics, illness severity and organ support for those eligible to receive a postal survey and those responding at either 3 or 12 months. The two groups were remarkably balanced in terms of their baseline demographics/measured confounders. Additional file 3 includes identical demographics with columns for those not responding to the study, explicitly removing consent and responding with and without symptoms of psychopathological issues.

**Table 1:** Baseline demographics, illness severity scores, organ support durations and admission diagnoses for those sent a questionnaire and patients responding

	Sent a questionnaire		Responders	
Patients (n)	13,155		4943	
Age (median) [25th–75th]	63 [47–73]		64 [52–73]	
Sex (%male)	57%		57%	
APACHE II score (median) [25th–75th]	15 [11–19]		15 [11–19]	
ICU length of stay (median) [25th–75th]	3 [2–6]		3 [2–6]	
Hospital length of stay (median) [25th–75th]	15 [8–29]		15 [9–28]	
Advanced cardiovascular support (% of patients)	23%		26%	
Basic cardiovascular support (% of patients)	90%		91%	
Advanced respiratory support (% of patients)	55%		56%	
Basic respiratory support (% of patients)	63%		64%	
Renal support (% of patients)	9%		10%	
Neurological support (% of patients)	10%		9%	
Liver support (% of patients)	0%		0%	
Dermatological support (% of patients)	4%		5%	
Gastrointestinal support (% of patients)	42%		43%	
Respiratory tract infection	*n* = 1152	9%	*n* = 419	8%
Vascular procedure to major vessel	*n* = 881	7%	*n* = 399	8%
Large bowel tumour	*n* = 695	5%	*n* = 315	6%
Self-poisoning	*n* = 572	4%	*n* = 117	2%
Septicaemia and septic shock	*n* = 431	3%	*n* = 173	3%
Acute renal failure	*n* = 473	4%	*n* = 169	3%
COPD	*n* = 393	3%	*n* = 155	3%
Malignant neoplasm of oesophagus	*n* = 272	2%	*n* = 145	3%
Status epilepticus	*n* = 318	2%	*n* = 80	2%
Bowel perforation	*n* = 305	2%	*n* = 106	2%
Acute pancreatitis	*n* = 192	1%	*n* = 83	2%
Diabetic ketoacidosis	*n* = 217	2%	*n* = 57	1%
Asthma attack in new or known asthmatic patients	*n* = 201	2%	*n* = 83	2%
Ventricular tachycardia or fibrillation	*n* = 169	1%	*n* = 75	2%
Acute myocardial infarction	*n* = 175	1%	*n* = 66	1%

### Outcome data

**Fig. 1:**
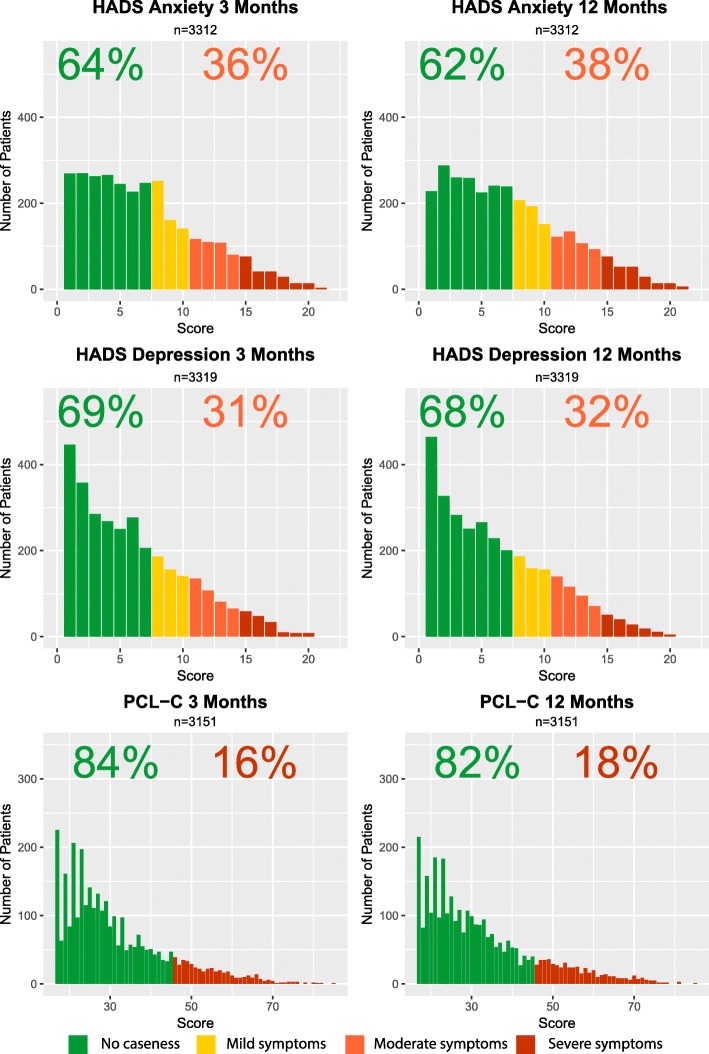
Caseness distribution against time for the Hospital Anxiety and Depression Score (HADS) and Post-Traumatic Stress Disorder Check List - Civilian (PCL-C) at 3 and 12 months post ICU discharge

Figure 1 shows the distribution of HADS anxiety, HADS depression and PCL-C in patients that provided valid responses at both 3 and 12 months. Percentages denote those with and without caseness. At 3 months the caseness prevalence for anxiety, depression and PTSD were 45.7%, 41.0% and 22.0% respectively. An identical analysis including all those responding at either time point is available in Additional file 4. Responses to individual questions are found in Additional files 5 and 6.

Whilst population prevalence is largely unchanged between 3 and 12 months across all three instruments, 10% of responders met the threshold for significant symptoms consistent with anxiety or depression at 12 months, who had not fulfilled these criteria when they responded at 3 months (Additional file 7). Similarly, 7% of responders met the symptom threshold for PTSD by 12 months, who had not when responding at 3 months. Therefore, between one third and one half of the patients meeting the threshold for caseness do so at only one of the two time points.

**Fig. 2:**
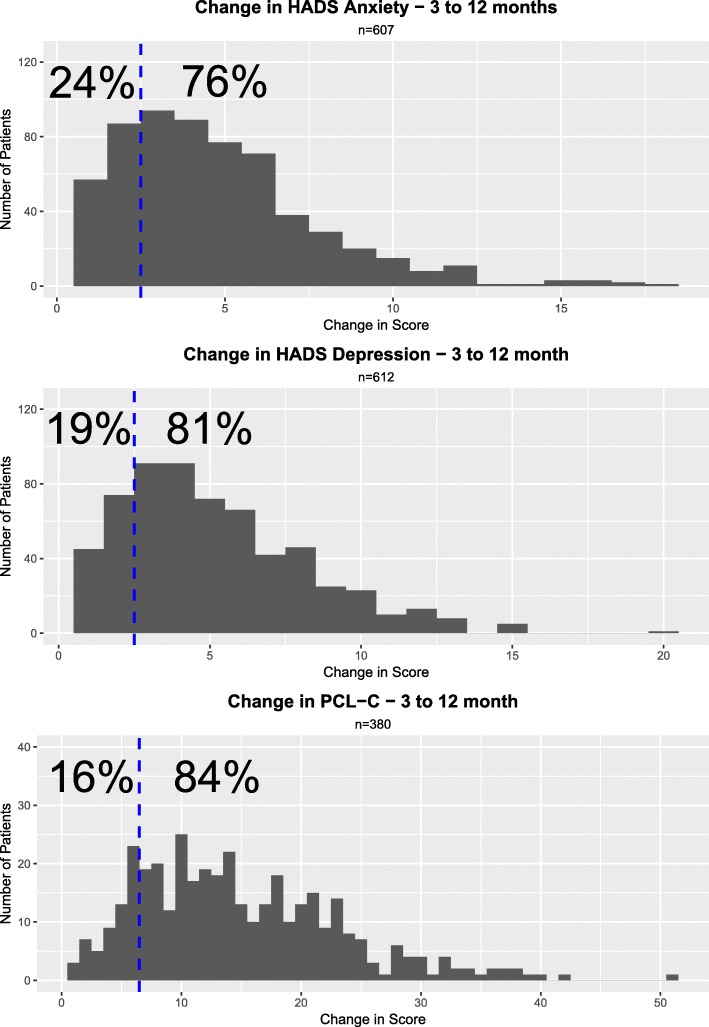
Analysis of change in the Hospital Anxiety and Depression Score (HADS)/Post-Traumatic Stress Disorder Check List - Civilian (PCL-C) in responders between 3 and 12 months post ICU discharge

Change analysis was performed for each instrument, including only those patients who reached the caseness threshold at one but not both time points (Fig. 2). The magnitude of this change was significant in the majority of cases: 76% and 81% of patients changed their anxiety/depression score by more than 3 points (3/22 patients (14% change)). A further 84% of patients experienced a change of 7 of more points (7/69 patients (10% change)) in their PCL-C score.

### Concurrent psychopathology

**Fig. 3:**
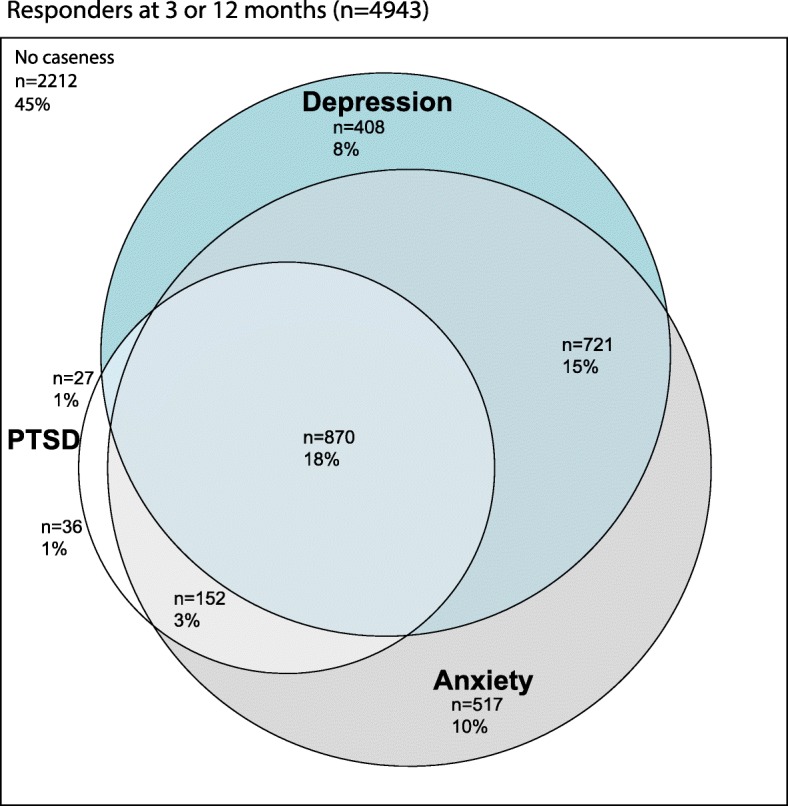
Cross over in caseness - anxiety/depression and post-traumatic stress disorder (PTSD) caseness amongst responders

Figure 3 shows the concurrent caseness of anxiety, depression and PTSD in individual patients. Of respondents, 55.2% (2731/4943) met caseness thresholds for at least one of the three conditions at either 3 or 12 months: 35.8% (1770/4943) met caseness thresholds for more than one psychopathological issue. Meeting caseness thresholds for PTSD alone was the least common (36 participants, 0.7% of those reporting psychopathological issues).

### Survival

**Fig. 4:**
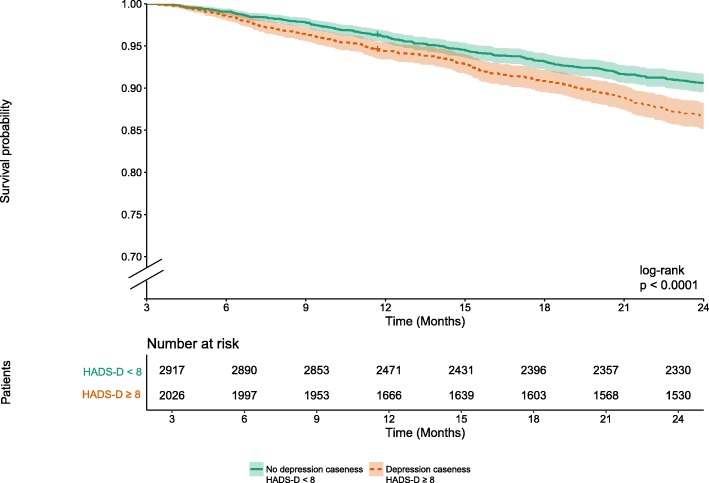
Survival versus depression (2 years) - Kaplan-Meier curve of 2-year survival amongst responders grouped by depression caseness. HADS, Hospital Anxiety and Depression Score

Figure 4 shows the 24-month survival of patients who responded at 3 or 12 months, dichotomised by those who had reported caseness for depression at either time point and those that had not. Survival data to 24 months were available for patients in phase 1 and 2 of the study, whilst phase 3 mortality data ended at 12 months (Additional file 8). Patients with a HADS-D ≥ 8 experienced a higher likelihood of death in the first 2 years after discharge from ICU (*p* < 0.001). Kaplan-Meier (KM) curves for anxiety and PTSD caseness are presented in Additional files 9 and 10 and do not demonstrate a significant difference between the respective groups.

**Fig. 5:**
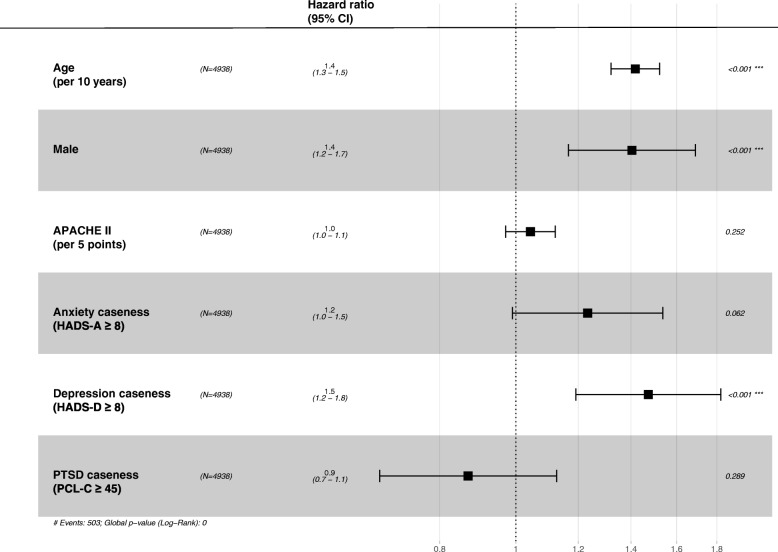
Hazard ratios showing the effect of known confounders (age, sex and illness severity), and anxiety, depression or post-traumatic stress disorder (PTSD) caseness on survival. APACHE, Acute Physiology and Chronic Health Evaluation; PCL-C, Post-Traumatic Stress Disorder Check List - Civilian

Results from the univariable and multivariable Cox proportional hazards modelling are reported in Additional file 11. Multivariable modelling was conducted for each type of caseness individually (models 1–3) and for all simultaneously (model 4). The hazard ratios in the multivariable models have been adjusted for all other variables in the model. Figure 5 is a graphical representation of the hazard ratios and confidence intervals from the final multivariable model (Additional file 11, model 4). This model takes the fact that some patients may demonstrate symptoms for one or more of anxiety, depression or PTSD into account (as seen in Fig. 3). Adjusted for age, sex, illness severity and the presence of other psychopathological issues, those meeting caseness thresholds for depression were around 50% more likely to die in the 2 years following discharge than those not meeting caseness (hazard ratio 1.47, 95% CI 1.19–1.80).

## Discussion

### Key results

We present the largest multicentre postal survey of self-reported anxiety, depression and PTSD of ICU survivors to date. A high burden of post-ICU psychopathological issues was reported with over half of respondents meeting caseness thresholds for anxiety, depression or PTSD. A high degree of symptom concurrency between these three conditions was observed. Especially interesting is the relatively low incidence of isolated PTSD, occurring in fewer than 1 in 20 individuals reporting any form of suspected psychopathological issue. Conversely, symptoms of anxiety occurred in 83% of individuals reporting any psychopathological issue. A strong association between self-reported symptoms of depression and mortality has been demonstrated in ICU survivors. When other known associations are adjusted for (age, male sex, illness severity), patients with depressive symptoms are nearly 50 % more likely to die during the first 24 months after leaving ICU than those without depression caseness.

### Interpretation and generalisability

Our study has shown that symptoms of anxiety, depression or PTSD occur in half of patients who respond to a postal questionnaire after treatment on a UK ICU, with nearly a third displaying symptoms of two or more conditions at 3 or 12 months following ICU discharge in the UK. This is comparable with incidence of persistent psychopathological issues in longer-term follow-up studies of survivors of ARDS where both the overall incidence of anxiety, depression and PTSD and co-occurrence of psychopathological issues are similar [26]. It is noteworthy that this pattern and prevalence of psychopathological issues extends across a UK general adult ICU population. Clinicians involved in the follow up and assessment of all survivors of ICU should be aware of the co-occurrence of psychopathological conditions as part of PICS. In this cohort, PTSD rarely occurs in isolation, instead strongly co-occurring with anxiety. Pre-existing anxiety has been demonstrated as a risk factor for developing PTSD in urban populations [27]; on meta-analysis a pre-existing psychopathological condition was the only pre-ICU factor that was consistently associated with PTSD symptoms. Conversely, PTSD symptomatology is strongly correlated with both post-ICU anxiety [3] and co-morbid depression post critical illness [28]. It seems logical that anxiety can lead to PTSD. However, these data lack any detail on pre-existing psychological status.

Our study discovered a previously unknown association between symptoms of depression and increased mortality for 2 years following ICU discharge. The association persists when adjusted for age, illness severity, sex and the presence of other psychopathological issues. Although depressive symptomatology is associated with increased mortality in other populations, it is with less magnitude than the 47% increased risk we have demonstrated following ICU discharge. For comparison, a large US study in a general veterans’ population estimates that diagnosed depression is associated with 17% greater hazard of all-cause mortality at 3 years [11]. This study also demonstrates that depression is associated with higher mortality rates for common medical conditions such as heart disease, respiratory illness, cerebrovascular disease, accidents, diabetes mellitus and hypertension. It remains unclear whether clinicians are overlooking the screening and treatment of depression in favour of treating chronic medical conditions or whether there is a biological association between chronic illness and depression. In the post-ICU population, the observed association between depression and mortality could be in part explained by the severity of chronic illness both pre-discharge and post-discharge - factors we did not adjust for in this study.

However, to the best of our knowledge, an association between depression and an increased rate of mortality after discharge from ICU has not been demonstrated previously. Depression could be a factor not previously considered in post-ICU survival. Given the incidence of depression amongst survivors, symptoms of this condition should be detected and managed during the time following ICU in primary care and in ICU follow-up clinics.

### Limitations

The main limitation of our postal survey is the relatively low response rate that potentially limits the applicability of these results. It is not possible using these data to infer the reason for non-response and as a consequence there is a potential selection/information bias in the results This postal survey followed best practice guidance and an RCT performed as part of this study demonstrated that a reduced questionnaire burden did not improve the response rate [16]. Failure of the postal network to return questionnaires is highly unlikely, however we cannot exclude the possibility that patients discharged from hospital may not initially return to their permanent address, limiting the delivery of questionnaires to recipients [29]. However, it is unlikely to be the universal reason. A significant proportion (22%) received a postal questionnaire and were able to contact the study in order to explicitly decline consent. There is clearly a significant group of people that are physically and mentally capable of returning a questionnaire but explicitly do not wish to take part in this form of research.

We suggest that the limited response rate is predominantly a methodological limitation of undertaking a postal survey in a post critical illness population in the UK. KM survival analysis of the 3–24-month period post ICU discharge included those meeting the caseness threshold at 3 or 12 months. There is the potential that including the 12-month respondents added bias. Reporting depression at 12 months would exclude those who had died in the preceding period and bias towards fewer deaths in those with depression.

A postal survey can only be used to calculate the prevalence of disease caseness rather than true clinical diagnostic rates. The validity of our findings is therefore reliant on the psychometric properties of the instruments used. The instruments employed here have been validated in similar cohorts. HADS [30] has been found to perform well in assessing the symptom severity and caseness of anxiety disorders and depression in patients with somatic symptoms and patients in psychiatric or primary care as well as in the general population [18, 31]. The psychometric properties of HADS have been evaluated in survivors of acute lung injury and its use has been suggested as part of a core-outcomes set for future clinical trials [32, 33]. Interpretation is based primarily on the cut-off scores. The authors of HADS suggested a 4-tier system (normal <= 7, mild 8–10, moderate 11–14 and severe 15–21) [19]. Subsequent studies have suggested a score of ≥ 8 provides sensitivity and specificity of 0.80 for both HADS anxiety and depression [34] although, certain conditions are known to affect the sensitivity and specificity e.g. traumatic brain injury [35].

This study had limited access to pre-morbid conditions, specifically pre-existing psychological and psychiatric conditions. Patients with pre-existing psychopathological conditions are at higher risk of both developing new symptoms and worsening existing problems following treatment in the ICU. In addition, it is possible that a phenotype exists where pre-morbid sufferers of anxiety/depression/PTSD are at a higher risk of developing critical illness. Future studies should collect pre-morbid psychological history in order to explore this hypothesis further.

The existing literature reports on the use of a variety of PTSD instruments, with the most popular being the Impact Event Scale (IES) [25]. Our study chose PCL-C above IES as the latter lacks sufficient sensitivity and specificity in the ICU population [36, 37]. Correlation of 0.93 between the total PCL-C score and structured interview with the Clinician-Administered PTSD Scale (CAPS) has been demonstrated (diagnostic efficiency 0.9) [38]. A score of 45 or greater on the PCL-C has been recommended as a cut-off for high PTSD symptom load, with sensitivity of 0.60 and specificity of 0.99 for diagnosing PTSD when compared with a structured clinical interview for the Diagnostic and Statistical Manual of Mental Health Disorders (DSM-IV) in survivors of breast cancer [20].

This study reports results from 3 different phases of the ICON study. There were small differences in the methodologies between studies (for a summary see Additional file 1). However, recruitment criteria and the instruments used to assess the psychopathological issues were identical, minimising the risks of including all 3 phases in this analysis.

### Future considerations

Following these results, future work should focus on describing the population that fails to respond to postal questionnaire. This should include determining whether study participants are in long-term care facilities, home care or otherwise incapacitated, as well as exploring some of the reasons why those who are physically and mentally able to respond choose not to. In addition, the utility of the postal questionnaire in detecting undiagnosed psychopathological issues should be evaluated and the treatments that result from detection described. This study strongly emphasises that future work should evaluate all forms of psychopathological conditions simultaneously, rather than focusing on a single specific condition. Collecting data on pre-morbid psychological and medical co-morbidities would also be essential in terms of understanding the risk factors for developing PICS, as current illness severity scoring and organ support information is clearly insufficient when it comes to understanding which individuals are at greatest risk.

## Conclusions

Half of those who respond to postal questionnaire following treatment on ICU in the UK report significant symptoms of anxiety, depression or PTSD at 3 and 12 months after discharge. When symptoms of one psychological condition are present, there is a 64% chance they will co-occur with symptoms of another. Survivors of critical illness who report symptoms of depression have an increased risk of dying in the 2 years following discharge from ICU.

## Additional files

Additional file 1: ICON study phases and survey breakdown. (DOCX 16 kb)

Additional file 2: Patient flow diagram. (PDF 196 kb)

Additional file 3: Demographics by subgroups of response, non-response and psychopathological burden. (DOCX 16 kb)

Additional file 4: Unlinked HADS/PCL-C pane. (PDF 192 kb)

Additional file 5: HADS responses. (DOCX 18 kb)

Additional file 6: PCL-C responses. (DOCX 17 kb)

Additional file 7: Changes in caseness against time - contingency tables showing the number and percentage of responders meeting the respective caseness thresholds at 3 and 12 months post ICU discharge. (DOCX 14 kb)

Additional file 8: KM - entire population. (PDF 152 kb)

Additional file 9: KM - anxiety. (PDF 159 kb)

Additional file 10: KM - PTSD. (PDF 156 kb)

Additional file 11: Cox proportional hazard regression analysis on factors affecting 2-year survival. (DOCX 14 kb)

## Abbreviations

APACHE: Acute Physiology and Chronic Health Evaluation
ARDS: Acute respiratory distress syndrome
EQ-5D-3 L: EuroQol group 5 Dimensions 3 level
HADS: Hospital Anxiety and Depression Score
ICNARC: Intensive Care National Audit & Research Centre
ICON: Intensive Care Outcome Network
ICU: Intensive care unit
MRIS: Medical Research Information Service
NHS: National Health Service
NIHR: National Institute for Health Research
ONS: Office of National Statistics
PCL-C: Post-Traumatic Stress Disorder Check List – Civilian
PTSD: Post-traumatic stress disorder
REC: Research Ethics Committee
SF36v2: Short Form 36 version 2
SWAT: Study within a trial
UK: United Kingdom
