# Supplementary material for: Anxiety, Depression and Post Traumatic Stress Disorder after critical illness: a UK-wide prospective cohort study
Source: Crit Care. 2018 Nov 23;22:310. doi: 10.1186/s13054-018-2223-6 (PMC6251214; doi:10.1186/s13054-018-2223-6)
Supplement: Supplementary file 1 — ICON study phases and survey breakdown. (DOCX 16 kb) [file 13054_2018_2223_MOESM1_ESM.docx]

# ICON Study Phases

|  | Phase 1 | Phase 2 | | Phase 3 |
| --- | --- | --- | --- | --- |
|  |  | **Group A (Alpha)** | **Group B (Beta)** |  |
| Instruments included in Questionnaire at 3 Months | | | | |
| VAS Prior | X | X | X | X |
| VAS Today | X | X | X | X |
| EQ-5D-3L | X | X | X | X |
| PCL-C | X |  | X | X |
| HADS (A/D) | X |  | X | X |
| SF36v2 | X |  |  | X |
| Demographics | X | X | X | X |
| Total pages |  |  |  |  |
| Instruments included in Questionnaire at 12 Months | | | | |
| VAS Prior | X | X | X | X |
| VAS Today | X | X | X | X |
| EQ-5D-3L | X | X | X | X |
| PCL-C | X |  | X | X |
| HADS (A/D) | X |  | X | X |
| SF36v2 | X |  |  | X |
| Demographics | X | X | X | X |
| Total pages |  |  |  |  |
| Instruments included in Questionnaire at 24 Months | | | | |
| VAS Prior |  |  |  |  |
| VAS Today | X | X | X |  |
| EQ-5D-3L | X | X | X |  |
| PCL-C |  |  |  |  |
| HADS (A/D) |  |  |  |  |
| SF36v2 |  |  |  |  |
| Demographics | X | X | X |  |
| Total pages |  |  |  |  |
| Study Details | | | | |
| Recruitment period | November 2006 to  May 2008 | May 2008 to October 2010 | | May 2012 to  May 2013 |
| Patients recruited | 9,582 | 18,490 | | 2,876 |
| Health Research Authority approval  (formally PIAG/NIGB) | Section 60 of the Health and Social Care Act 2001: **PIAG 2-05(e)/2006**  (subsequently Section 251 of the NHS Act 2006) | | | |
| Ethical Approval | Oxfordshire Research Ethics Committee B  **REC 06/Q1605/17** | | | NRES - South Central Berkshire  **REC 11/SC/0172** |
