# Supplementary figures and images for: Anxiety, Depression and Post Traumatic Stress Disorder after critical illness: a UK-wide prospective cohort study
Source: Crit Care. 2018 Nov 23;22:310. doi: 10.1186/s13054-018-2223-6 (PMC6251214; doi:10.1186/s13054-018-2223-6)

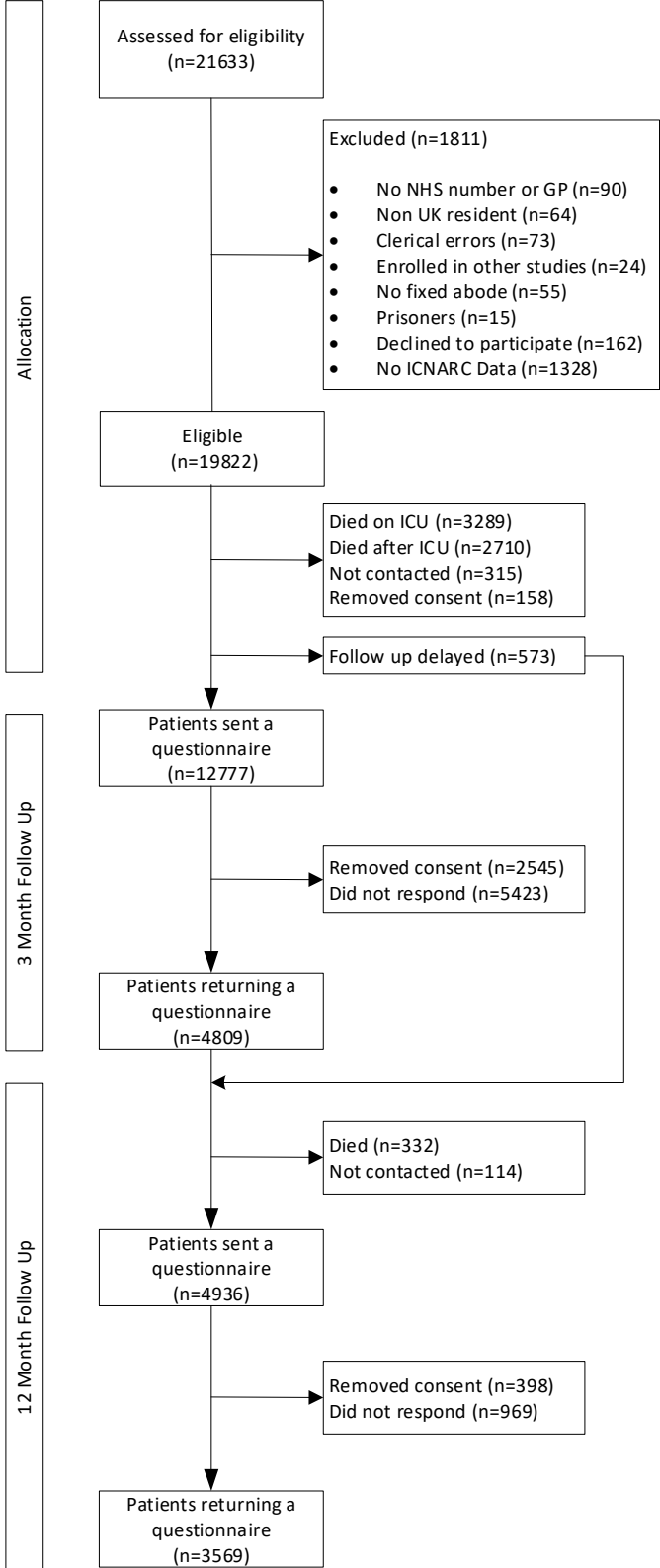

Supplement: Supplementary file 2 — Patient flow diagram. (PDF 196 kb) [file 13054_2018_2223_MOESM2_ESM.pdf]

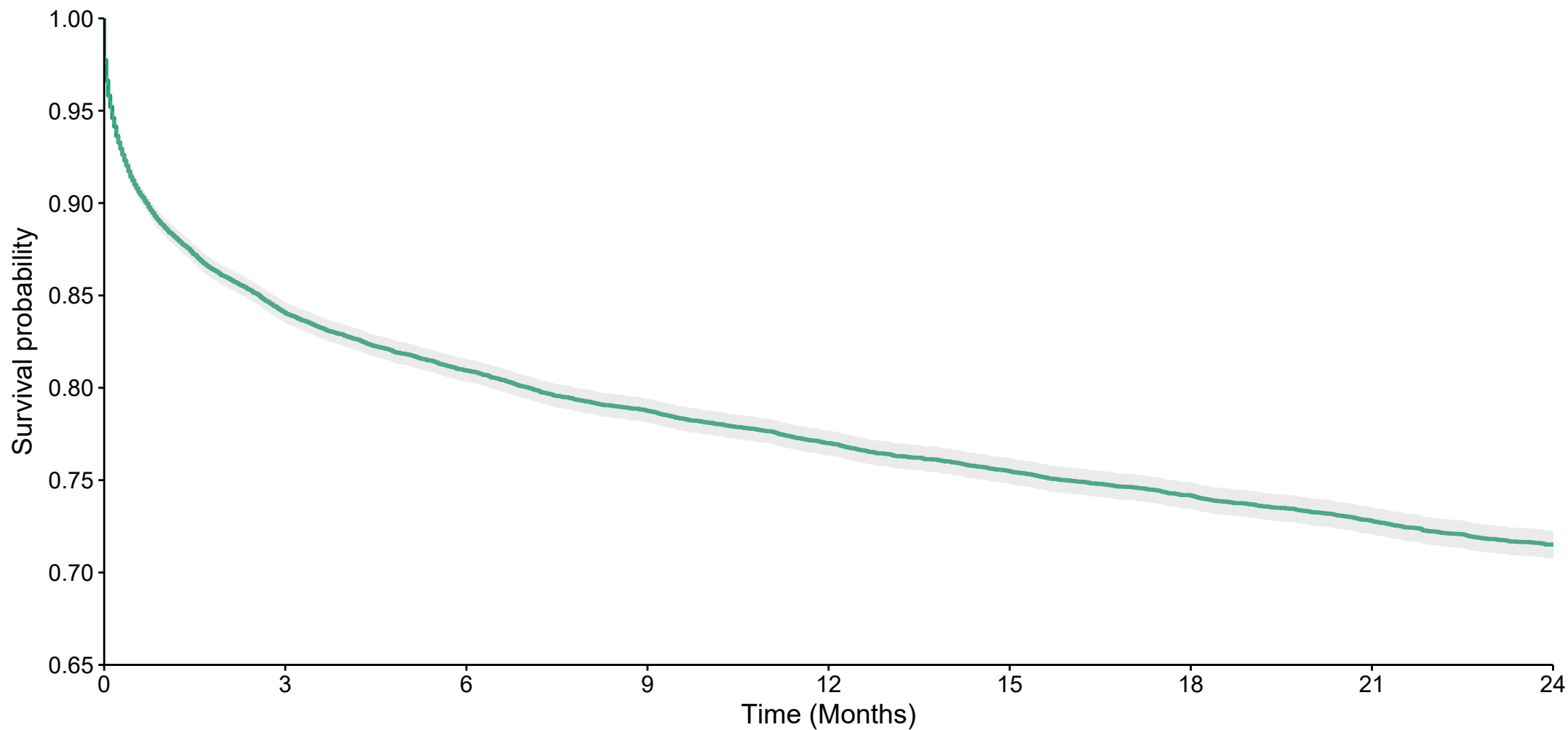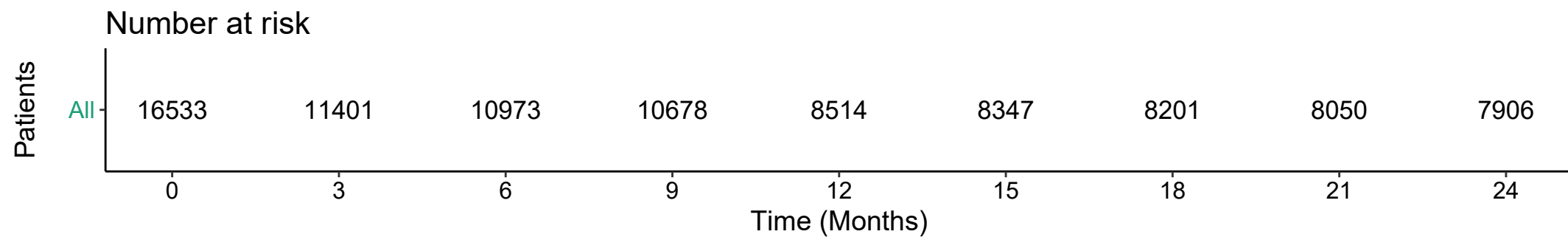

Supplement: Supplementary file 8 — KM - entire population. (PDF 152 kb) [file 13054_2018_2223_MOESM8_ESM.pdf]
