# Supplementary material for: Anxiety, Depression and Post Traumatic Stress Disorder after critical illness: a UK-wide prospective cohort study
Source: Crit Care. 2018 Nov 23;22:310. doi: 10.1186/s13054-018-2223-6 (PMC6251214; doi:10.1186/s13054-018-2223-6)
Supplement: Supplementary file 3 — Demographics by subgroups of response, non-response and psychopathological burden. (DOCX 16 kb) [file 13054_2018_2223_MOESM3_ESM.docx]

|  | **Missing data: All** | **Missing data: No contact from patient** | **Missing data: Explicitly removed consent** | **Responders: All** | **Responders: No psychopathology** | **Responders: Anxiety caseness** | **Responders: Depression caseness** | **Responders: PTSD caseness** |
| --- | --- | --- | --- | --- | --- | --- | --- | --- |
| **Patients n** | 8212 | 5591 | 2621 | 4943 | 2212 | 2260 | 2026 | 1085 |
| **Age (median) [25th-75th]** | 61 [ 44 - 73 ] | 56 [ 40 - 70 ] | 70 [ 59 - 78 ] | 64 [ 52 - 73 ] | 67 [ 57 - 75 ] | 60 [ 47 - 70 ] | 62 [ 49 - 72 ] | 55 [ 43 - 65 ] |
| **Sex (% male)** | 57 % | 57 % | 57 % | 57 % | 62 % | 51 % | 56 % | 50 % |
| **APACHE II score (median) [25th-75th]** | 14 [ 11 - 19 ] | 14 [ 10 - 19 ] | 15 [ 12 - 19 ] | 15 [ 11 - 19 ] | 15 [ 11 - 19 ] | 14 [ 11 - 19 ] | 15 [ 11 - 19 ] | 14 [ 10 - 19 ] |
| **ICU length of stay (median) [25th-75th]** | 3 [ 2 - 6 ] | 3 [ 2 - 6 ] | 3 [ 2 - 5 ] | 3 [ 2 - 6 ] | 3 [ 2 - 6 ] | 3 [ 2 - 7 ] | 3 [ 2 - 7 ] | 4 [ 2 - 8 ] |
| **Hospital Length of stay (median) [25th-75th]** | 15 [ 8 - 30 ] | 15 [ 7 - 30 ] | 16 [ 9 - 31 ] | 15 [ 9 - 28 ] | 14 [ 9 - 25 ] | 15 [ 9 - 29 ] | 17 [ 9 - 31 ] | 16 [ 9 - 29 ] |
| **Advanced cardiovascular support (% of patients)** | 17 % | 17 % | 18 % | 22 % | 23 % | 20 % | 20 % | 19 % |
| **Basic cardiovascular support (% of patients)** | 75 % | 76 % | 74 % | 78 % | 77 % | 77 % | 78 % | 79 % |
| **Advanced respiratory support (% of patients)** | 46 % | 48 % | 41 % | 47 % | 46 % | 48 % | 49 % | 53 % |
| **Basic respiratory support (% of patients)** | 52 % | 52 % | 50 % | 55 % | 52 % | 57 % | 58 % | 59 % |
| **Renal support (% of patients)** | 8 % | 8 % | 7 % | 9 % | 8 % | 9 % | 10 % | 11 % |
| **Neuro support (% of patients)** | 8 % | 9 % | 6 % | 8 % | 7 % | 9 % | 9 % | 9 % |
| **Liver support (% of patients)** | 0 % | 1 % | 0 % | 0 % | 0 % | 0 % | 0 % | 0 % |
| **Dermatological supports (% of patients)** | 3 % | 4 % | 3 % | 4 % | 3 % | 4 % | 4 % | 5 % |
| **Gastro intestinal support (% of patients)** | 34 % | 35 % | 31 % | 37 % | 34 % | 38 % | 40 % | 42 % |
|  | Respiratory tract infection n= 733 9 % | Respiratory tract infection n= 522 9 % | Vascular procedure to major vessel n= 255 10 % | Respiratory tract infection n= 419 8 % | Vascular procedure to major vessel n= 248 11 % | Respiratory tract infection n= 204 9 % | Respiratory tract infection n= 188 9 % | Respiratory tract infection n= 106 10 % |
|  | Vascular procedure to major vessel n= 482 6 % | Self poisoning n= 370 7 % | Respiratory tract infection n= 211 8 % | Vascular procedure to major vessel n= 399 8 % | Large bowel tumour n= 183 8 % | Large bowel tumour n= 110 5 % | Vascular procedure to major vessel n= 103 5 % | Self poisoning n= 67 6 % |
|  | Self poisoning n= 455 6 % | Vascular procedure to major vessel n= 227 4 % | Large bowel tumour n= 189 7 % | Large bowel tumour n= 315 6 % | Respiratory tract infection n= 166 8 % | Vascular procedure to major vessel n= 105 5 % | Large bowel tumour n= 92 5 % | Septicaemia and septic shock n= 39 4 % |
|  | Large bowel tumour n= 380 5 % | Acute renal failure n= 204 4 % | Acute renal failure n= 100 4 % | Septicaemia and septic shock n= 173 3 % | Septicaemia and septic shock n= 78 4 % | Self poisoning n= 90 4 % | Acute renal failure n= 88 4 % | Vascular procedure to major vessel n= 39 4 % |
|  | Acute renal failure n= 304 4 % | Large bowel tumour n= 191 3 % | COPD n= 87 3 % | Acute renal failure n= 169 3 % | Malignant neoplasm of oesophagus n= 77 3 % | COPD n= 83 4 % | Self poisoning n= 80 4 % | COPD n= 35 3 % |
|  | Septicaemia and septic shock n= 258 3 % | Status n= 189 3 % | Self poisoning n= 85 3 % | COPD n= 155 3 % | Acute renal failure n= 64 3 % | Acute renal failure n= 82 4 % | COPD n= 77 4 % | Acute renal failure n= 34 3 % |
|  | COPD n= 238 3 % | Septicaemia and septic shock n= 186 3 % | Bowel perforation n= 74 3 % | Malignant neoplasm of oesophagus n= 145 3 % | COPD n= 56 3 % | Septicaemia and septic shock n= 78 3 % | Septicaemia and septic shock n= 71 4 % | Asthma attack in new or known asthmatic n= 32 3 % |
|  | Status n= 238 3 % | COPD n= 151 3 % | NA n= 73 3 % | Self poisoning n= 117 2 % | Ventricular tachycardia or fibrillation n= 46 2 % | Malignant neoplasm of oesophagus n= 55 2 % | Bowel perforation n= 56 3 % | Large bowel tumour n= 32 3 % |
|  | Bowel perforation n= 199 2 % | Diabetic ketoacidosis n= 135 2 % | Septicaemia and septic shock n= 72 3 % | Bowel perforation n= 106 2 % | Acute pancreatitis n= 37 2 % | Bowel perforation n= 54 2 % | Malignant neoplasm of oesophagus n= 48 2 % | Bowel perforation n= 27 2 % |
|  | Diabetic ketoacidosis n= 160 2 % | Bowel perforation n= 125 2 % | Status n= 49 2 % | Acute pancreatitis n= 83 2 % | Bowel perforation n= 34 2 % | Asthma attack in new or known asthmatic n= 47 2 % | Status n= 43 2 % | Status n= 25 2 % |

Appendix K
