# Supplementary material for: Anxiety, Depression and Post Traumatic Stress Disorder after critical illness: a UK-wide prospective cohort study
Source: Crit Care. 2018 Nov 23;22:310. doi: 10.1186/s13054-018-2223-6 (PMC6251214; doi:10.1186/s13054-018-2223-6)
Supplement: Supplementary file 4 — Unlinked HADS/PCL-C pane. (PDF 192 kb) [file 13054_2018_2223_MOESM4_ESM.pdf]

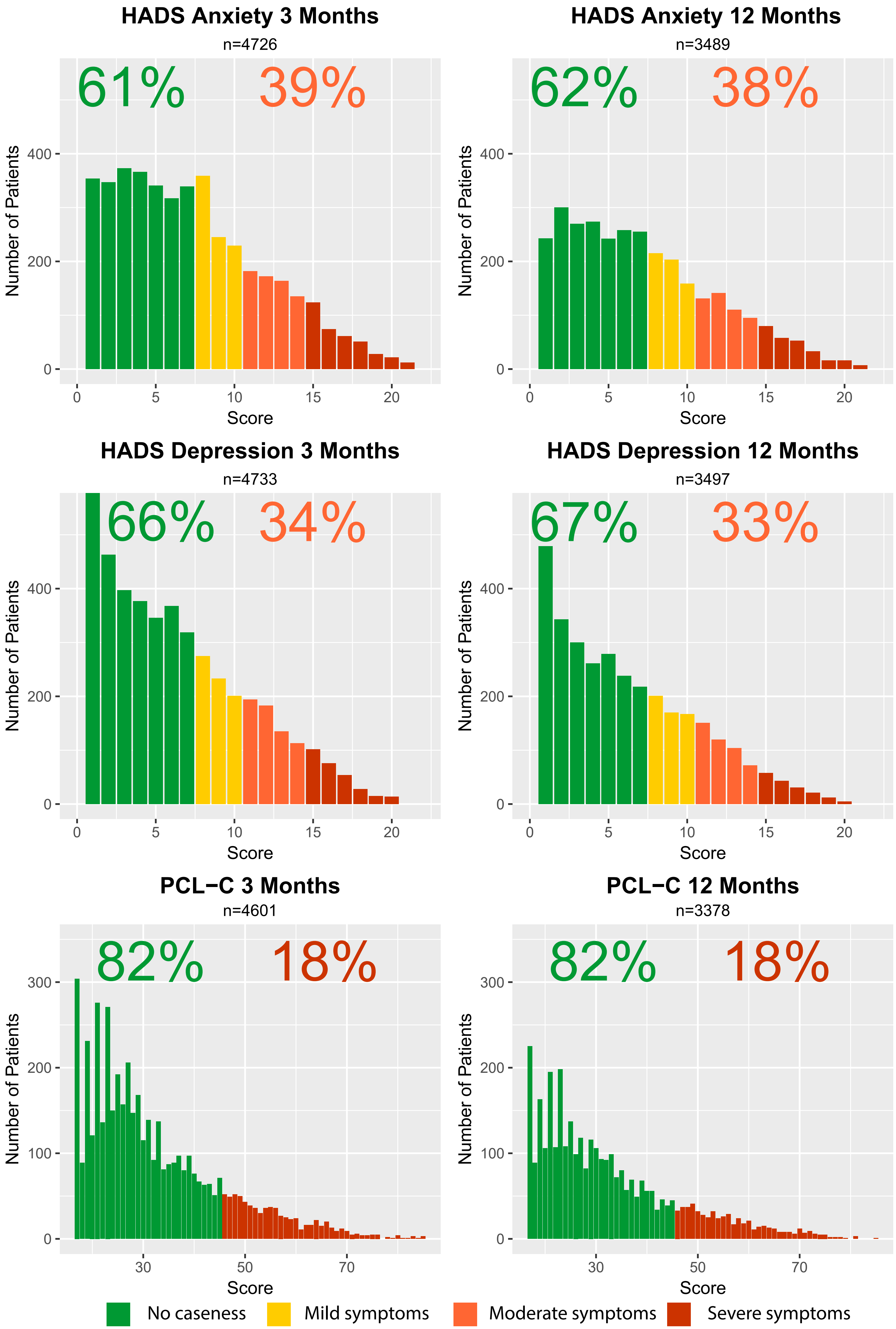
HADS Anxiety 3 Months
n=4726
61%
39%
HADS Anxiety 12 Months
n=3489
62%
38%
HADS Depression 3 Months
n=4733
66%
34%
HADS Depression 12 Months
n=3497
67%
33%
PCL−C 3 Months
n=4601
82%
18%
PCL−C 12 Months
n=3378
82%
18%
Number of Patients
Score
No caseness
Mild symptoms
Moderate symptoms
Severe symptoms
