# Supplementary material for: Anxiety, Depression and Post Traumatic Stress Disorder after critical illness: a UK-wide prospective cohort study
Source: Crit Care. 2018 Nov 23;22:310. doi: 10.1186/s13054-018-2223-6 (PMC6251214; doi:10.1186/s13054-018-2223-6)
Supplement: Supplementary file 5 — HADS responses. (DOCX 18 kb) [file 13054_2018_2223_MOESM5_ESM.docx]

**HADS Anxiety**

|  | **3 months** | **12 months** |
| --- | --- | --- |
| **Patients n** | 3312 | 3312 |
|  | | |
| **I feel tense or 'wound up'** | | |
| 0 - Not at all | 1058 (32%) | 961 (29%) |
| 1 - From time to time, occasionally | 1679 (51%) | 1710 (52%) |
| 2 - A lot of the time | 426 (13%) | 479 (14%) |
| 3 - Most of the time | 161 (5%) | 186 (6%) |
|  | | |
| **I get a sort of frightened feeling as if something awful is about to happen** | | |
| 0 - Not at all | 1365 (41%) | 1317 (40%) |
| 1 - A little, but it doesn't worry me | 991 (30%) | 1020 (31%) |
| 2 - Yes, but not too badly | 717 (22%) | 739 (22%) |
| 3 - Very definitely and quite badly | 254 (8%) | 275 (8%) |
|  | | |
| **Worrying thoughts go through my mind** | | |
| 0 - Only occasionally | 1234 (37%) | 1182 (36%) |
| 1 - From time to time, but not too often | 1198 (36%) | 1207 (36%) |
| 2 - A lot of the time | 600 (18%) | 595 (18%) |
| 3 - A great deal of the time | 325 (10%) | 364 (11%) |
|  | | |
| **I can sit at ease and feel relaxed** | | |
| 0 - Definitely | 1048 (32%) | 1025 (31%) |
| 1 - Usually | 1543 (47%) | 1530 (46%) |
| 2 - Not Often | 627 (19%) | 686 (21%) |
| 3 - Not at all | 109 (3%) | 110 (3%) |
|  | | |
| **I get a sort of frightened feeling like 'butterflies' in the stomach** | | |
| 0 - Not at all | 1692 (51%) | 1576 (48%) |
| 1 - Occasionally | 1233 (37%) | 1284 (39%) |
| 2 - Quite Often | 298 (9%) | 359 (11%) |
| 3 - Very Often | 107 (3%) | 114 (3%) |
|  | | |
| **I feel restless as I have to be on the move** | | |
| 0 - Not at all | 1085 (33%) | 1052 (32%) |
| 1- Not very much | 1171 (35%) | 1235 (37%) |
| 2 - Quite a lot | 891 (27%) | 865 (26%) |
| 3 - Very much indeed | 189 (6%) | 187 (6%) |
|  | | |
| **I get sudden feelings of panic** | | |
| 0 - Not at all | 1627 (49%) | 1523 (46%) |
| 1 - Not very often | 1046 (32%) | 1110 (34%) |
| 2 - Quite often | 506 (15%) | 539 (16%) |
| 3 - Very often indeed | 139 (4%) | 154 (5%) |

**HADS Depression**

|  | **3 months** | **12 months** |
| --- | --- | --- |
| **Patients n** | 3319 | 3319 |
|  | | |
| **I still enjoy the things I used to enjoy** | | |
| 0 - Definitely as much | 1098 (33%) | 1105 (33%) |
| 1 - Not quite so much | 1372 (41%) | 1427 (43%) |
| 2 - Only a little | 458 (14%) | 478 (14%) |
| 3 - Hardly at all | 406 (12%) | 342 (10%) |
|  | | |
| **I can laugh and see the funny side of things** | | |
| 0 - As much as I always could | 2092 (63%) | 2066 (62%) |
| 1 - Not quite so much now | 876 (26%) | 895 (27%) |
| 2 - Definitely not so much now | 306 (9%) | 331 (10%) |
| 3 - Not at all | 48 (1%) | 45 (1%) |
|  | | |
| **I feel cheerful** | | |
| 0 - Most of the time | 1910 (58%) | 1896 (57%) |
| 1 - Sometimes | 1060 (32%) | 1080 (33%) |
| 2 - Not often | 302 (9%) | 298 (9%) |
| 3 - Not at all | 65 (2%) | 66 (2%) |
|  | | |
| **I feel as if I am slowed down** | | |
| 0 - Not at all | 284 (9%) | 328 (10%) |
| 1 - Sometimes | 1140 (34%) | 1217 (37%) |
| 2 - Very often | 889 (27%) | 852 (26%) |
| 3 - Nearly all the time | 1045 (31%) | 961 (29%) |
|  | | |
| **I have lost interest in my appearance** | | |
| 0 - I take just as much care as ever | 1971 (59%) | 1849 (56%) |
| 1 - I may not take quite as much care | 796 (24%) | 844 (25%) |
| 2 - I don't take as much care as I should | 442 (13%) | 516 (16%) |
| 3 - Definitely | 125 (4%) | 124 (4%) |
|  | | |
| **I look forward with enjoyment to things** | | |
| 0 - As much as I ever did | 1614 (49%) | 1545 (47%) |
| 1 - Rather less than I used to | 1072 (32%) | 1091 (33%) |
| 2 - Definitely less than I used to | 164 (5%) | 171 (5%) |
| 3 - Hardly at all | 478 (14%) | 536 (16%) |
|  | | |
| **I can enjoy a good book/radio/tv program** | | |
| 0 - Often | 2298 (69%) | 2257 (68%) |
| 1 - Sometimes | 750 (23%) | 795 (24%) |
| 2 - Not often | 163 (5%) | 185 (6%) |
| 3 - Very seldom | 129 (4%) | 103 (3%) |

**Appendix D – HADS responses at 3 and 12 months**
