# Supplementary material for: Anxiety, Depression and Post Traumatic Stress Disorder after critical illness: a UK-wide prospective cohort study
Source: Crit Care. 2018 Nov 23;22:310. doi: 10.1186/s13054-018-2223-6 (PMC6251214; doi:10.1186/s13054-018-2223-6)
Supplement: Supplementary file 6 — PCL-C responses. (DOCX 17 kb) [file 13054_2018_2223_MOESM6_ESM.docx]

|  | **3 months** | **12 months** |
| --- | --- | --- |
| **Patients n** | 3151 | 3151 |
|  |  |  |
| **Repeated, disturbing memories** | | |
| Not at all | 1398 (44%) | 1341 (43%) |
| A little bit | 509 (16%) | 610 (19%) |
| Moderately | 829 (26%) | 803 (25%) |
| Quite a bit | 288 (9%) | 271 (9%) |
| Extremely | 151 (5%) | 154 (5%) |
|  | | |
| **Repeated, disturbing dreams** | | |
| Not at all | 2388 (76%) | 2314 (73%) |
| A little bit | 312 (10%) | 368 (12%) |
| Moderately | 301 (10%) | 307 (10%) |
| Quite a bit | 101 (3%) | 110 (3%) |
| Extremely | 65 (2%) | 68 (2%) |
|  | | |
| **Flashbacks** | | |
| Not at all | 2450 (78%) | 2372 (75%) |
| A little bit | 301 (10%) | 326 (10%) |
| Moderately | 293 (9%) | 315 (10%) |
| Quite a bit | 84 (3%) | 109 (3%) |
| Extremely | 51 (2%) | 65 (2%) |
|  | | |
| **Feeling very upset** | | |
| Not at all | 2397 (76%) | 2245 (71%) |
| A little bit | 288 (9%) | 347 (11%) |
| Moderately | 333 (11%) | 375 (12%) |
| Quite a bit | 101 (3%) | 129 (4%) |
| Extremely | 52 (2%) | 71 (2%) |
|  | | |
| **Physical reactions** | | |
| Not at all | 2567 (81%) | 2521 (80%) |
| A little bit | 17 (1%) | 29 (1%) |
| Moderately | 492 (16%) | 525 (17%) |
| Quite a bit | 17 (1%) | 28 (1%) |
| Extremely | 126 (4%) | 160 (5%) |
|  | | |
| **Avoid thinking about a stressful experience** | | |
| Not at all | 2210 (70%) | 2139 (68%) |
| A little bit | 210 (7%) | 256 (8%) |
| Moderately | 358 (11%) | 356 (11%) |
| Quite a bit | 246 (8%) | 283 (9%) |
| Extremely | 155 (5%) | 157 (5%) |
|  | | |
| **Avoid activities or situations** | | |
| Not at all | 2538 (81%) | 2437 (77%) |
| A little bit | 175 (6%) | 204 (6%) |
| Moderately | 216 (7%) | 249 (8%) |
| Quite a bit | 151 (5%) | 210 (7%) |
| Extremely | 99 (3%) | 123 (4%) |
|  | | |
| **Trouble remembering** | | |
| Not at all | 1271 (40%) | 1309 (42%) |
| A little bit | 263 (8%) | 265 (8%) |
| Moderately | 585 (19%) | 592 (19%) |
| Quite a bit | 391 (12%) | 421 (13%) |
| Extremely | 729 (23%) | 656 (21%) |
|  | | |
| **Loss of interest** | | |
| Not at all | 1563 (50%) | 1519 (48%) |
| A little bit | 280 (9%) | 311 (10%) |
| Moderately | 744 (24%) | 739 (23%) |
| Quite a bit | 359 (11%) | 398 (13%) |
| Extremely | 261 (8%) | 268 (9%) |
|  | | |
| **Feeling distant** | | |
| Not at all | 1888 (60%) | 1812 (58%) |
| A little bit | 255 (8%) | 254 (8%) |
| Moderately | 602 (19%) | 643 (20%) |
| Quite a bit | 251 (8%) | 275 (9%) |
| Extremely | 171 (5%) | 195 (6%) |
|  | | |
| **Feeling emotionally numb** | | |
| Not at all | 2375 (75%) | 2293 (73%) |
| A little bit | 31 (1%) | 35 (1%) |
| Moderately | 697 (22%) | 759 (24%) |
| Quite a bit | 31 (1%) | 35 (1%) |
| Extremely | 141 (4%) | 169 (5%) |
|  | | |
| **Feeling as if your future will somehow be cut short** | | |
| Not at all | 1725 (55%) | 1578 (50%) |
| A little bit | 14 (0%) | 19 (1%) |
| Moderately | 1056 (34%) | 1158 (37%) |
| Quite a bit | 14 (0%) | 19 (1%) |
| Extremely | 398 (13%) | 453 (14%) |
|  | | |
| **Trouble falling or staying asleep** | | |
| Not at all | 1610 (51%) | 1577 (50%) |
| A little bit | 12 (0%) | 12 (0%) |
| Moderately | 989 (31%) | 998 (32%) |
| Quite a bit | 12 (0%) | 12 (0%) |
| Extremely | 576 (18%) | 600 (19%) |
|  | | |
| **Feeling irritable or having angry outbursts** | | |
| Not at all | 1914 (61%) | 1772 (56%) |
| A little bit | 11 (0%) | 15 (0%) |
| Moderately | 1018 (32%) | 1111 (35%) |
| Quite a bit | 11 (0%) | 15 (0%) |
| Extremely | 241 (8%) | 298 (9%) |
|  | | |
| **Having difficulty concentrating** | | |
| Not at all | 1643 (52%) | 1646 (52%) |
| A little bit | 6 (0%) | 12 (0%) |
| Moderately | 1183 (38%) | 1177 (37%) |
| Quite a bit | 6 (0%) | 12 (0%) |
| Extremely | 337 (11%) | 352 (11%) |
|  | | |
| **Being “super alert”** | | |
| Not at all | 2356 (75%) | 2316 (74%) |
| A little bit | 17 (1%) | 18 (1%) |
| Moderately | 645 (20%) | 682 (22%) |
| Quite a bit | 17 (1%) | 17 (1%) |
| Extremely | 184 (6%) | 186 (6%) |
|  | | |
| **Feeling jumpy or easily startled** | | |
| Not at all | 2090 (66%) | 1985 (63%) |
| A little bit | 11 (0%) | 11 (0%) |
| Moderately | 762 (24%) | 828 (26%) |
| Quite a bit | 11 (0%) | 11 (0%) |
| Extremely | 321 (10%) | 360 (11%) |

**Appendix E – PCL-C responses at 3 and 12 months**
