# Supplementary material for: Anxiety, Depression and Post Traumatic Stress Disorder after critical illness: a UK-wide prospective cohort study
Source: Crit Care. 2018 Nov 23;22:310. doi: 10.1186/s13054-018-2223-6 (PMC6251214; doi:10.1186/s13054-018-2223-6)
Supplement: Supplementary file 7 — Changes in caseness against time - contingency tables showing the number and percentage of responders meeting the respective caseness thresholds at 3 and 12 months post ICU discharge. (DOCX 14 kb) [file 13054_2018_2223_MOESM7_ESM.docx]

| **Anxiety Caseness** | | **12 months** | |  |
| --- | --- | --- | --- | --- |
|  |  | **-ve** | **+ve** | **Total** |
| **3 months** | **-ve** | 1790  **54%** | 335  **10%** | 2125  **64%** |
|  | **+ve** | 272  **8%** | 915  **28%** | 1187  **36%** |
|  | **Total** | 2062  **62%** | 1250  **38%** | 3312 |

**Table 2: Change in Anxiety caseness against time**

**Table 3: Change in Depression caseness against time**

| **Depression Caseness** | | **12 months** | |  |
| --- | --- | --- | --- | --- |
|  |  | **-ve** | **+ve** | **Total** |
| **3 months** | **-ve** | 1955  **59%** | 326  **10%** | 2281  **69%** |
|  | **+ve** | 286  **9%** | 752  **22%** | 1038  **31%** |
|  | **Total** | 2241  **68%** | 1078  **32%** | 3319 |

**Table 4: Change in PTSD caseness against time**

| **PTSD Caseness** | | **12 months** | |  |
| --- | --- | --- | --- | --- |
|  |  | **-ve** | **+ve** | **Total** |
| **3 months** | **-ve** | 2419  **77%** | 223  **7%** | 2642  **84%** |
|  | **+ve** | 157  **5%** | 352  **11%** | 509  **16%** |
|  | **Total** | 2576  **82%** | 575  **18%** | 3151 |
