# Supplementary material for: Anxiety, Depression and Post Traumatic Stress Disorder after critical illness: a UK-wide prospective cohort study
Source: Crit Care. 2018 Nov 23;22:310. doi: 10.1186/s13054-018-2223-6 (PMC6251214; doi:10.1186/s13054-018-2223-6)
Supplement: Supplementary file 9 — KM - anxiety. (PDF 159 kb) [file 13054_2018_2223_MOESM9_ESM.pdf]

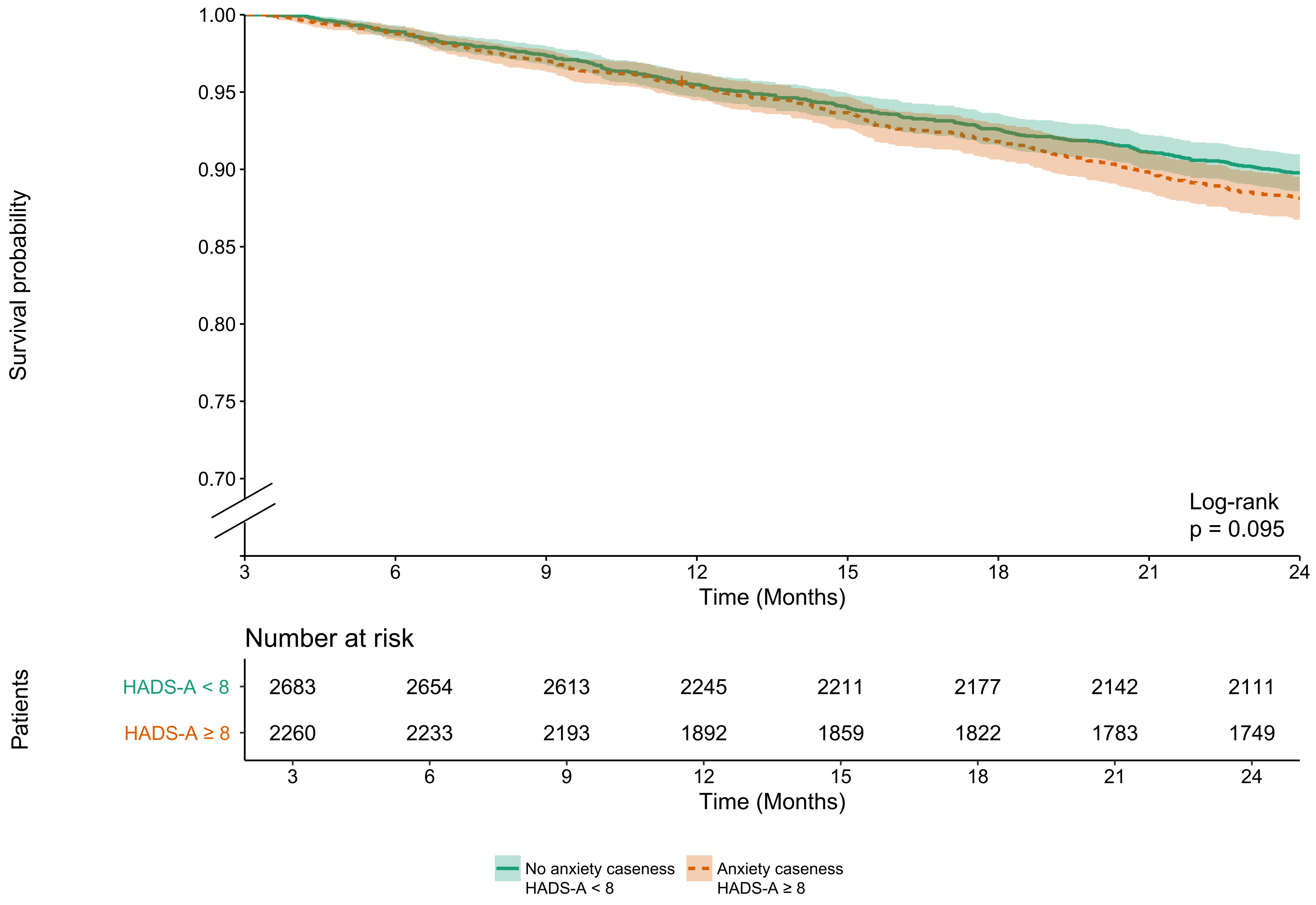

Survival probability
1.00
0.95
0.90
0.85
0.80
0.75
0.70
Log-rank
p = 0.095
3
6
9
12
15
18
21
24
Time (Months)
Number at risk
Patients
HADS-A < 8
2683
2654
2613
2245
2211
2177
2142
2111
HADS-A ≥ 8
2260
2233
2193
1892
1859
1822
1783
1749
Time (Months)
No anxiety caseness
HADS-A < 8
Anxiety caseness
HADS-A ≥ 8
