# Supplementary material for: Anxiety, Depression and Post Traumatic Stress Disorder after critical illness: a UK-wide prospective cohort study
Source: Crit Care. 2018 Nov 23;22:310. doi: 10.1186/s13054-018-2223-6 (PMC6251214; doi:10.1186/s13054-018-2223-6)
Supplement: Supplementary file 10 — KM - PTSD. (PDF 156 kb) [file 13054_2018_2223_MOESM10_ESM.pdf]

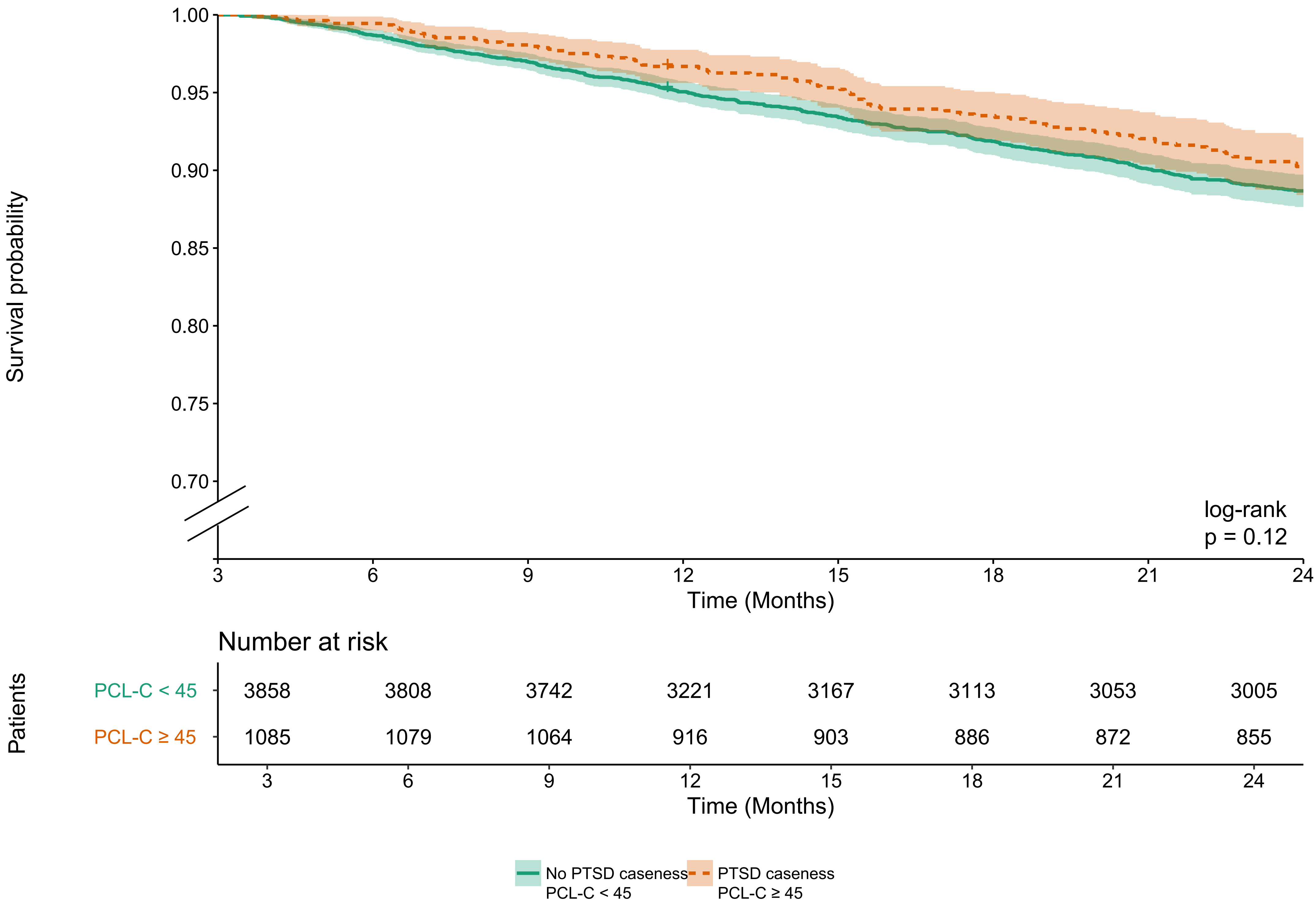

Survival probability
1.00
0.95
0.90
0.85
0.80
0.75
0.70
log-rank
p = 0.12
3
6
9
12
15
18
21
24
Time (Months)
Number at risk
Patients
PCL-C < 45
3858
3808
3742
3221
3167
3113
3053
3005
PCL-C ≥ 45
1085
1079
1064
916
903
886
872
855
3
6
9
12
15
18
21
24
Time (Months)
No PTSD caseness
PCL-C < 45
PTSD caseness
PCL-C ≥ 45
