# Supplementary material for: Anxiety, Depression and Post Traumatic Stress Disorder after critical illness: a UK-wide prospective cohort study
Source: Crit Care. 2018 Nov 23;22:310. doi: 10.1186/s13054-018-2223-6 (PMC6251214; doi:10.1186/s13054-018-2223-6)
Supplement: Supplementary file 11 — Cox proportional hazard regression analysis on factors affecting 2-year survival. (DOCX 14 kb) [file 13054_2018_2223_MOESM11_ESM.docx]

Hazard ratios for association between two-year mortality and age, male, apache 2 score and caseness for anxiety, depression and PTSD

|  | **Characteristic** | **Hazard ratio (95% CI)** | | | | |
| --- | --- | --- | --- | --- | --- | --- |
|  |  | **Univariable models** | **Multivariable model 1*** | **Multivariable model 2*** | **Multivariable model 3*** | **Multivariable model 4*** |
|  | **Age**  **(per 10 years)** | 1.42 (1.33-1.51) | 1.43 (1.33-1.53) | 1.42 (1.32-1.52) | 1.42 (1.32-1.52) | 1.42 (1.32-1.52) |
|  | **Male** | 1.48 (1.23-1.78) | 1.42 (1.18-1.71) | 1.38 (1.15-1.66) | 1.37 (1.14-1.65) | 1.40 (1.17-1.69) |
|  | **APACHE II**  **(per 5 points)** | 1.14 (1.07-1.22) | 1.05 (0.97-1.13) | 1.04 (0.97-1.12) | 1.05 (0.98-1.13) | 1.04 (0.97-1.12) |
| **Caseness** | **Anxiety**  **(HADS-A ≥8)** | 1.16 (0.97-1.38) | 1.45 (1.22-1.74) |  |  | 1.23 (0.99-1.54) |
|  | **Depression**  **(HADS-D ≥8)** | 1.45 (1.21-1.72) |  | 1.58 (1.32-1.88) |  | 1.47 (1.19-1.82) |
|  | **PTSD**  **(PCL-C ≥45)** | 0.84 (0.67-1.05) |  |  | 1.21 (0.96-1.52) | 0.87 (0.67-1.13) |

*Each risk factor (age, male, apache 2 score, anxiety, depression and PTSD caseness) has been adjusted for the other factors. Multivariable modelling was performed for each form of caseness (anxiety, depression, PTSD) individually (model 1-3) and in combination (model 4).
